# Pharmacogenetic Biomarkers to Predict Treatment Response in Multiple Sclerosis: Current and Future Perspectives

**DOI:** 10.1155/2017/6198530

**Published:** 2017-07-19

**Authors:** Patricia K. Coyle

**Affiliations:** Department of Neurology and MS Comprehensive Care Center, Stony Brook University Medical Center, Stony Brook, NY 11794, USA

## Abstract

Disease-modifying therapies (DMTs) have significantly advanced the treatment of relapsing multiple sclerosis (MS), decreasing the frequency of relapses, disability, and magnetic resonance imaging lesion formation. However, patients' responses to and tolerability of DMTs vary considerably, creating an unmet need for biomarkers to identify likely responders and/or those who may have treatment-limiting adverse reactions. Most studies in MS have focused on the identification of pharmacogenetic markers, using either the candidate-gene approach, which requires prior knowledge of the genetic marker and its role in the target disease, or genome-wide association, which examines multiple genetic variants, typically single nucleotide polymorphisms (SNPs). Both approaches have implicated numerous alleles and SNPs in response to selected MS DMTs. None have been validated for use in clinical practice. This review covers pharmacogenetic markers in clinical practice in other diseases and then reviews the current status of MS DMT markers (interferon *β*, glatiramer acetate, and mitoxantrone). For a complex disease such as MS, multiple biomarkers may need to be evaluated simultaneously to identify potential responders. Efforts to identify relevant biomarkers are underway and will need to be expanded to all MS DMTs. These will require extensive validation in large patient groups before they can be used in clinical practice.

## 1. Introduction

Multiple sclerosis (MS) is a chronic, immune-mediated, inflammatory, neurodegenerative disorder. It is the leading nontraumatic cause of disability in young adults and is estimated to affect >100/100,000 inhabitants in North America and Europe [1, 2]. The diagnosis is three times more common in women than in men [1, 2]. The precise etiology of MS is unknown; however, there is strong evidence that it arises from complex interactions between environmental and genetic factors. With regard to genetic factors, genome-wide association studies (GWAS) have identified almost 200 genetic variants associated with MS susceptibility, most of which are involved in the immune response and are often associated with other immune-mediated diseases [3–6]. A strong association has been found between the major histocompatibility complex (MHC) region and MS susceptibility, with human leukocyte antigen- (HLA-) DRB1^*∗*^1501 showing the strongest effect [7]. Other contributors include the interleukin-2 receptor and interleukin-7 receptor alleles [8]. Environmental factors associated with MS include exposure to infectious organisms, for example, Epstein-Barr virus, vitamin D levels and sunlight exposure, tobacco use, geographical latitude, and possibly antigenic determinants in the gut microbiome [9–12].

Although there are currently no therapies recognized to reverse the neurodegenerative process of MS, significant progress has been made over the last two decades in the treatment of relapsing forms of MS (RMS) with the introduction of disease-modifying therapies (DMTs) that decrease the frequency of relapses and slow development of disability. Available DMTs used in patients with RMS have diverse mechanisms of action. They include interferon betas (IFN*β*s: intramuscular IFN*β*-1a, subcutaneous [SC] IFN*β*-1a, SC IFN*β*-1b, and SC pegylated IFN*β*-1a), glatiramer acetate, mitoxantrone, alemtuzumab, natalizumab, daclizumab, fingolimod, teriflunomide, and dimethyl fumarate [13]. Responses to these treatments, as defined by reduced relapse rates, improved magnetic resonance imaging outcomes, and preservation of neurological function, vary between patients, and there are notable differences in adverse effect profiles [14]. For example, there is evidence for associations between treatment with IFN*β* and increased liver enzyme levels and, occasionally, with serious liver injury. Glatiramer acetate treatment can cause localized lipoatrophy that can be distressing to patients. Natalizumab treatment, and to a much lesser extent dimethyl fumarate and fingolimod treatments, is associated with a rare but potentially fatal demyelinating disorder known as progressive multifocal leukoencephalopathy. Mitoxantrone treatment can cause cardiotoxicity and acute leukemia. Fingolimod has been associated with herpes infections, macular edema, and cardiac conduction issues. Although attempts are underway to identify biomarkers that can be used to monitor response to therapy during treatment [15, 16] and patients are monitored for the occurrence of adverse effects, it would clearly be beneficial if biomarkers could be used before initiating treatment to identify patients who are likely to respond. It would also be helpful to identify those who are more likely to experience a serious adverse effect [14]. Such approaches would avoid unnecessary costs and negative effects on quality of life resulting from treating patients with drugs to which they will not respond and which may be associated with unacceptable adverse events.

Drug response is affected by pharmacokinetic, pharmacodynamic, physiological, pathological, and environmental factors; drug mechanism of action, formulation, and route of administration; and patient characteristics (Table 1). Genetic polymorphisms that influence the activity of proteins regulating the pharmacodynamic and pharmacokinetic properties of drugs are key contributors to the variability in response to drugs between individuals. For example, cytochrome P450 (CYP) enzymes are the major enzymes involved in drug metabolism. Polymorphisms in these enzymes can result in either ultrafast metabolism of therapeutic drugs, thereby limiting their efficacy, or poor metabolism, thereby increasing the risk of toxicity [17]. Identification of genetic differences associated with variability in drug response would allow better-informed decisions regarding choice of treatment. The aim of this article is to review basic definitions of genetic biomarkers and how they are being used in other disorders and then to review their current status in MS.

## 2. Pharmacogenetic Biomarkers

Pharmacogenomics is the study of how genes affect drug response. Both inherited and acquired genetic variations may be involved. Meaningful biomarkers allow physicians to tailor treatment to patients' individual genetic and genomic characteristics. Accordingly, a pharmacogenomic biomarker is defined as a measurable DNA and/or RNA characteristic that is an indicator of normal biologic processes, pathogenic processes, and/or response to therapeutic or other interventions [18]. A genomic biomarker could, for example, be the degree of expression of a gene, the function of a gene, or the regulation of a gene [18]. Pharmacogenetics is a subset of pharmacogenomics. It involves variations in DNA sequences as they relate to drug metabolism and response [18]. A genetic variation may range from a single nucleotide polymorphism (SNP) to loss of part of a chromosome. Key applications for pharmacogenetic biomarkers are the identification of responders and nonresponders to medications, avoidance of adverse events, and optimization of drug dose.

### 2.1. Pharmacogenetic Biomarkers in Practice

There are currently over 150 US Food and Drug Administration- (FDA-) approved drugs with pharmacogenomic information in their labeling [19]. Almost all therapeutic areas have at least one drug for which pharmacogenomic guidance exists, including psychiatry, rheumatology, gastroenterology, endocrinology, and dermatology; however, by far, the best represented therapeutic area is oncology (Table 2). The vast majority of drugs have guidance concerning variations in DNA sequence which relate to drug response, which are therefore classed as pharmacogenetic biomarkers. Examples of drugs with pharmacogenetic guidance are presented in Table 3. Pharmacogenetic information may pertain to many aspects of drug use, including definition of specific patient populations for which a drug is indicated or contraindicated, for which dose adjustments could be necessary, and also in which potentially serious adverse events could occur. For the majority of oncology drugs, the inclusion of biomarkers in drug labels generally corresponds to a requirement or recommendation for genetic testing; however, in other therapeutic areas, there is no specific guidance on what actions should be taken based on biomarker information.

In the area of infectious diseases, there are at least 17 drugs with pharmacogenetic information in their labeling [19]. For the majority of these, the guidance relates to the presence of a pharmacogenetic marker and the increased likelihood of adverse effects associated with treatment. Abacavir is a synthetic carbocyclic nucleoside analog with inhibitory activity against human immunodeficiency virus. The labeling of abacavir states that all patients should be screened for the presence of the HLA-B^*∗*^5701 allele before initiating or reinitiating abacavir therapy, unless the patient has a previously documented HLA-B^*∗*^5701 allele assessment [20]. Abacavir is contraindicated in patients who are positive for the HLA-B^*∗*^5701 allele because of the high risk of experiencing a hypersensitivity reaction [20]. Systematic analysis has indicated that testing for HLA-B^*∗*^5701 before initiating abacavir is cost-effective [21], and companion diagnostic tests are available for this allele, although no specific test is recommended in the drug label [22].

In oncology, there are at least 54 drugs with pharmacogenetic information in their labeling [19]. For over half, the pharmacogenetic information relates to a specific indication or usage [19]. Vemurafenib is a kinase inhibitor that was specifically designed to inhibit mutated serine/threonine-protein kinase B-Raf (*BRAF*), in patients with metastatic melanoma who harbor the *BRAF*^V600E^ mutation. It is not recommended for use in patients with wild-type* BRAF* melanoma, as safety and efficacy have not been demonstrated in this population. Accordingly, the requirement for the presence of this mutation is specified in the indications for vemurafenib within the drug label [23]. In the first example of its kind, a companion diagnostic test for vemurafenib, the cobas® 4800 BRAF V600 Mutation Test (Roche Molecular Systems, Inc., Pleasanton, CA, USA), was developed and received FDA approval alongside the drug. Despite the codevelopment and coapproval of the drug and diagnostic test, the label does allow for another FDA-approved test to be used if desired [23].

Only a handful of hematology drugs have pharmacogenetic information in their labeling, mostly relating to warnings and precautions [19]. Lenalidomide is one of the few drugs with pharmacogenetic information pertaining to its indication. In an initial study involving patients with myelodysplastic syndromes who did not respond to treatment with recombinant erythropoietin, a greater proportion of patients with a 5q deletion no longer needed red cell transfusions after being treated with lenalidomide compared with patients with other karyotypes [24]. This observation was confirmed in further studies, leading to a defined indication for lenalidomide for the treatment of transfusion-dependent anemia due to International Prognostic Scoring System low- or intermediate-1 risk myelodysplastic syndromes associated with a 5q deletion abnormality with or without additional cytogenetic abnormalities [25]. No specific guidance is provided regarding testing for 5q deletion.

Some drugs used to treat heritable genetic diseases, such as cystic fibrosis and certain inborn errors of metabolism, also have pharmacogenetic information in their labeling. For example, ivacaftor, a cystic fibrosis transmembrane conductance regulator potentiator, is indicated for the treatment of cystic fibrosis in patients aged 2 years and older who have defined mutations in the* CFTR* gene (G551D, G1244E, G1349D, G178R, G551S, S1251N, S1255P, S549N, or S549R). According to the label, if the patient's genotype is unknown, an FDA-approved cystic fibrosis mutation test should be used to detect the presence of a* CFTR* mutation [26]. Specific guidelines are available to facilitate the interpretation of genotype tests to guide ivacaftor therapy [27].

In the field of schizophrenia, aripiprazole, an atypical antipsychotic, has pharmacogenetic information in its label relating to its use in patients with poor CYP2D6 metabolism. Aripiprazole is partly metabolized by CYP2D6 and exposure to the active moieties of the drug is ~60% higher in poor metabolizers of CYP2D6 substrates. In endocrinology, glimepiride, a sulfonylurea indicated as an adjunct to diet and exercise to improve glycemic control in adults with type 2 diabetes mellitus, has guidance in its label relating to its use in individuals who are glucose-6-phosphate dehydrogenase-deficient, owing to the risk of hemolytic anemia. Finally, in the area of transplantation, mycophenolic acid is contraindicated in patients with hereditary deficiency of hypoxanthine-guanine phosphoribosyltransferase, as use of mycophenolate in such patients may cause exacerbation of disease symptoms.

## 3. Approaches to Identifying Pharmacogenetic Biomarkers in Polygenic Diseases

To date, the routinely used pharmacogenetic biomarkers reflect relatively simple, well-defined genetic changes. Considerable efforts are now being made to establish pharmacogenetic biomarkers for polygenic diseases, such as cancer, chronic kidney disease, MS, cardiovascular disease, and neuropsychiatric illnesses. In these indications, numerous genes and their products are potentially involved in disease manifestation, drug metabolism, and drug mechanism of action, and the individual contribution of each gene may be small [28–30]. In 2008, a systematic review of pharmacogenetic studies found that most had examined genetic variations in drug targets (i.e., pharmacodynamic studies) rather than examining genes encoding proteins involved in drug handling and elimination (pharmacokinetic studies) [29]. However, this is no doubt a consequence of early studies using a candidate-gene approach to identify potential genes. The candidate-gene approach directly tests the effects of genetic variants of a potentially contributing gene in an association study. A higher frequency of a particular allele or genotype in a series of individuals with a specific disease or phenotype can be interpreted as meaning that the allelic variant or genotype is associated with that disease or the disease phenotype [31]. A key limitation of this approach is that it is dependent on knowledge of the biology of the disease being investigated in order to identify candidate genes for testing. In contrast, GWAS examine multiple genetic variants, typically SNPs, in different individuals to see if any variant is associated with a trait or response to a drug. The advantage of the GWAS approach is that it does not require specific knowledge of the disease in question; however, it is not without its own challenges. For example, effect sizes for common variants are typically modest and large sample sizes are required to detect associations; single genomic regions can harbor variants with weak effects and also large effects; some associations implicate non-protein-coding regions; and correlations between genetic variants and phenotypes can be limited by the accuracy and validity of the phenotypic measurement [32].

For pharmacogenetic information to be routinely used in clinical practice, the genetic markers will require validation in large cohorts, particularly in cases where response is found to be influenced by numerous genetic variants. Predictive diagnostic tests also require validation before use, and regulatory frameworks are evolving to ensure that diagnostic tests accurately identify the intended population for a corresponding treatment.

### 3.1. Pharmacogenetic Markers in MS

Over the last decade, considerable effort has been made to identify pharmacogenetic markers in the field of MS. To date, efforts have been focused on the identification of markers that determine drug response, and there are no published data relating to pharmacogenetic markers to predict adverse drug reactions. A review conducted in 2013 outlined a number of candidate genes for future study but noted the challenges in identification of pharmacogenetic markers for adverse drug reactions including the relative rarity of these reactions, the need for accurate characterization of the reaction, and accurate phenotyping of the patient [14].

Regarding pharmacogenetic markers for drug response, longitudinal data on drug response and disease worsening are necessary for their identification and potential validation. As a consequence, the majority of data relate to the established first-line agents IFN*β* and glatiramer acetate, with limited data on mitoxantrone. At the time of writing, there were no data available on pharmacogenetic markers for natalizumab or fingolimod. Although there are no universally accepted measures of response for DMTs, most studies evaluate a clinically event-free status, for example, relapse-free status with no confirmed worsening on the Expanded Disability Status Score, as a measure of a positive response.

#### 3.1.1. Interferon *β*

IFN*β* is a pleiotropic cytokine that mediates its effects through binding of the heterodimeric IFN*α*/*β* receptor, which activates Janus kinase- (JAK-) signal transducer and activator of transcription (STAT) signaling, and induces genes with multiple biological functions. The mechanism of action of IFN*β* in MS has not yet been fully elucidated. It is thought that IFN*β* reduces the inflammatory processes that lead to demyelination in MS, promoting a regulatory versus proinflammatory milieu through multiple actions, such as changing cytokine network balance, reducing antigen presentation and T cell proliferation, altering cytokine and matrix metalloproteinase expression, and restoring suppressor function [33]. Clinical trials show that treatment with IFN*β* is associated with prevention of relapse compared with placebo across all relapsing subtypes of MS [34–36]; however, up to 30–50% of patients show suboptimal responses to therapy [37–39]. As determination of response requires clinical follow-up for at least 2 years, this “wait and see” approach is clearly suboptimal as it can result in the delay of an alternative, potentially more effective treatment, being initiated. In addition, this approach unnecessarily exposes patients to potential adverse effects and can add to the substantial economic burden carried by healthcare providers. There is an unmet need for biomarkers that reliably correlate with response to IFN*β* in MS.

Pharmacogenetic biomarkers for IFN*β* response in MS have recently been extensively reviewed [39, 40]. Initial attempts used the candidate-gene approach, selecting genes with known associations with IFN*β* mechanisms and signaling pathways. Using this method, around 15 genes show significant associations with response to IFN*β* (Table 4) [39, 41–52]. Genes with polymorphisms associated with response to IFN*β* include those that encode the common receptor for type I IFNs, IFN response-element sequences, IFN regulatory transcription factors, the ubiquitin-specific peptidase gene, and other cytokine genes. For the majority of polymorphisms, significant associations with positive IFN*β* treatment response were found, although some were associated with nonresponse [39]. Not all of the significant associations have been validated, and further studies in large populations with independent validation are warranted. The validity of many candidate biomarkers was recently called into question in a study of ten “responders” and ten “nonresponders” with RMS who were retrospectively selected on the basis of stringent clinical criteria after a 5-year follow-up [53]. The baseline expression levels of 25 IFN-regulated genes (*MxA*,* GPR3*,* IL17RC*,* ISG15*,* TRAIL*,* OASL*,* IFIT1*,* IFIT2*,* RSAD2*,* OAS3*,* IFI44L*,* TRIM22*,* IL10*,* CXCL10*,* STAT1*,* OAS1*,* OAS2*,* IFNAR1*,* IFNAR2*,* IFNβ*,* ISG20*,* IFI6*,* PKR*,* IRF7*, and* USP18*) were examined but were not confirmed to have predictive value [53].

To date, five GWAS have been conducted to identify polymorphisms associated with IFN*β* response in MS [54–58]. These have been carried out in initial populations of between approximately 100 and 350 patients, with follow-up to determine response for up to 4 years. Although the findings of the earliest study were not independently validated, subsequent studies were validated in independent populations to further test their significance. Across the studies, a number of genes have been found to be positively associated with positive or negative response to IFN*β*. These genes and the potential function of the proteins they encode are summarized in Table 5 and include those for glypican 5 (*GPC5*) collagen type XXV alpha-1, hyaluronan proteoglycan link protein, calpastatin, and neuronal PAS domain protein 3 [54]; alpha-amino-3-hydroxy-5-methyl-4-isoxazolepropionic acid-type glutamate receptor GRIA3, type 1 IFN-related proteins ADAR and IFNAR2, cell cycle-dependent protein CIT, zinc finger proteins ZFAT and ZFHX4, and guanosine triphosphatase-activating protein STARD13 [56]; SLC9A9, an Na^+^/H^+^ exchanger found in endosomes [57];* NINJ2*,* TBXAS1*, and genes related to the glutamatergic system (*GRM3* and* GRIK2*) [55]; and* FHIT* (fragile histidine triad) and* GAPVD1* (GTPase-activating protein and VPS9 domains 1) [58]. Surprisingly, there has been little overlap across studies with regard to the genes that have been implicated as being significantly associated with treatment response. For example, the most recent study did not find an association for the genes for* GPC5* and* SLC9A9* [58], as was identified by two previous studies [54, 57].

The reasons underlying the lack of consistent findings across the studies are unclear but may reflect the lack of an unequivocal definition of responder status, small sample sizes, and different populations. It is also possible that the contribution of single alleles of candidate genes is very small and that combinations of alleles should be studied together to identify markers for therapeutic response. Multilocus analyses have been attempted and some have identified sets of alleles that show significant associations. For example, in an analysis of 61 SNPs in 34 candidate genes as possible determinants of IFN*β* response in Irish patients with MS, the most significant allelic combinations that differed in frequency between responders and nonresponders included* JAK2–IL10RB–GBP1–PIAS1*, followed by* JAK2–IL10–CASP3* [59]. It is perhaps not surprising that multiple genes will influence the efficacy of a drug with pleiotropic effects; it therefore seems likely that the multiallele approach will represent the most likely path to identify responders. As with all pharmacogenetic studies, it will be important to validate any findings in studies that include large numbers of patients with comprehensive clinical information to allow determination of drug-response status [58].

#### 3.1.2. Glatiramer Acetate

Glatiramer acetate is a mixture of synthetic polymers composed of random sequences of four amino acids, which simulates myelin basic protein. In MS, it exerts immunomodulatory effects and is proposed to have neuroprotective properties, although its precise mechanism of action is not known. Genome-wide expression studies show that it alters the expression of thousands of genes [60]. Glatiramer acetate appears to modulate the immune response by altering T cell differentiation, causing a shift from T cells with a proinflammatory T helper (Th)1/Th17 phenotype to T cells with the anti-inflammatory Th2/T-regulatory phenotype, which may dampen neighboring inflammation within the central nervous system [61, 62]. It also exerts immunomodulatory activity on antigen-presenting cells, such as macrophages and dendritic cells, which participate in innate immune responses [61].

As with IFN*β*, most data relating to the pharmacogenetics of glatiramer acetate have been identified through candidate-gene studies. In a recent review, ten genes with polymorphisms associated with glatiramer acetate response were identified from candidate-gene studies (Table 6) [39, 40, 44, 63–65]. The first genes identified with polymorphisms associated with glatiramer acetate treatment response were HLA class II genes [44, 63, 64]. This is in contrast to IFN*β* response, where no association was observed with the HLA class II genes [39]. Other candidate genes that have shown an association with response to glatiramer acetate include those involved in T cell activation and the formation of trimolecular complexes (T cell receptor, MHC molecule, and antigen) necessary for T lymphocytes to recognize antigen [39].

Only one GWAS has been conducted to date with glatiramer acetate [66]. This analysis, which was conducted on a subset of good and poor responders to glatiramer acetate, identified 11 SNPs, the majority of which related to genes involved in glatiramer acetate's mechanism of action. As with IFN*β*, attempts have also been made to identify groups of genes which influence response, taking into account the potentially pleiotropic effects of the drug and the likelihood that multiple genes influence its activity. Findings from this approach are showing promise. For example, in an analysis of nine polymorphisms in candidate genes in 285 Russian patients with MS, carriers of one combination of alleles, DRB1^*∗*^15 + TGFB1^*∗*^T + CCR5^*∗*^d + IFNAR1^*∗*^G and DRB1^*∗*^15 + TGFB1^*∗*^T + CCR5^*∗*^d, had a 14- to 15-fold increased risk of poor response to glatiramer acetate therapy. Similar to IFN*β*, it seems that the multiallele approach will represent the most likely path to identify responders. Again, any findings will require validation in studies of large MS populations with comprehensive clinical information.

#### 3.1.3. Mitoxantrone

Mitoxantrone is a synthetic anthracenedione derivative that exerts immunomodulatory effects in patients with MS, although these remain to be fully elucidated [67]. It intercalates into DNA and inhibits both DNA replication and RNA synthesis and blocks DNA repair. Clinical trial data indicate that intravenous mitoxantrone treatment improved neurological disability and delayed progression of MS in patients with worsening relapsing disease [68]. The impact of SNPs in the ATP-binding cassette transporters ABCB1 and ABCB2 on the efficacy of mitoxantrone was examined retrospectively in 155 patients with MS [69]. ATP-binding cassette transporters are transmembrane proteins influencing drug absorption, excretion, and the extent of drug entry into target organs. Analysis showed that the clinical response rate increased significantly with ATP-binding cassette transporter genotypes that were known to be associated with decreased function. As with all potential biomarkers, the findings require comprehensive validation in a large MS population.

## 4. Conclusions

Variability in response to DMTs in patients with MS represents a significant clinical challenge. Potentially, delays may occur in patients receiving a treatment to which they can optimally respond, exposing them to adverse effects without significant benefit and placing a huge burden on healthcare systems. Drug efficacy is affected by multiple genetic factors; it would be advantageous to understand those factors in order to identify patients who will respond to a particular drug before they receive it. Although there have been notable successes in identifying pharmacogenetic biomarkers for relatively simple, well-defined genetic changes in other diseases, the identification of markers for MS has been more challenging. Candidate-gene approaches have implicated numerous alleles with varying levels of significance, and GWAS have added to the wealth of data on the topic. For a complex disease such as MS, for which key treatments have pleiotropic effects that are not fully understood, it is becoming apparent that multiple biomarkers may need to be evaluated simultaneously to identify responders. Efforts to identify groups of relevant biomarkers are beginning to show associations with responses, but, based on current data, the routine use of pharmacogenetics in MS is not imminent. Central to research in this area will be the validation of any markers and groups of markers in large well-defined patient populations, with clear definitions of response and nonresponse criteria.

## Figures and Tables

**Table 1:** Major factors affecting drug response in patients.

Factor	Examples
Pharmacokinetic	Absorption, distribution, metabolism, and/or excretion of drugs

Pharmacodynamic	Disease state Availability of therapeutic targets, for example, the presence or absence of drug receptors

External factors	Environmental chemicals (including smoking) Coadministered drugs

Drug	Drug form Dosing frequency Route of administration Drug mechanism of action

Physiological	Age, weight, sex, pregnancy, race, and food consumption

Pathological	Liver disease Renal disease Malnutrition

Patient behavior	Compliance and persistence with drug regimens

**Table 2:** Therapy areas using therapies with pharmacogenomic information in their labels.

Therapy area	Number of drugs^*∗*^
Oncology	54
Psychiatry	27
Infectious diseases	17
Endocrinology	10
Neurology	9
Gastroenterology	9
Cardiology	8
Rheumatology	6
Inborn errors of metabolism	6
Hematology	5
Anesthesiology	4
Pulmonary	4
Urology	2
Dermatology	2
Dental	1
Gynecology	1
Transplantation	1
Toxicology	1

^*∗*^Search of http://www.fda.gov/Drugs/ScienceResearch/ResearchAreas/Pharmacogenetics/ucm083378.htm conducted on July 27, 2016.

**Table 3:** Examples of pharmacogenetic biomarkers in clinical practice.

Disease	Drug	Biomarker	Significance
HIV	Abacavir	HLA-B^*∗*^5701 allele	Presence of HLA-B^*∗*^5701 allele indicates high risk of hypersensitivity reaction

Metastatic melanoma	Vemurafenib	*BRAF* ^V600E^ mutation	Presence of *BRAF*^V600E^ mutation specified in drug indication

Myelodysplastic syndrome	Lenalidomide	5q deletion	Presence of 5q deletion specified in drug indication

Cystic fibrosis	Ivacaftor	Defined mutations in the *CFTR* gene (G551D, G1244E, G1349D, G178R, G551S, S1251N, S1255P, S549N, or S549R)	Presence of defined mutations allows drug use

Schizophrenia	Aripiprazole	CYP2D6	Dosage adjustments necessary in CYP2D6 poor metabolizers

Transplantation	Mycophenolic acid	HGPRT	Contraindicated in patients with hereditary deficiency of HGPRT

Type 2 diabetes mellitus	Glimepiride	G6PD	Can cause hemolytic anemia in patients with G6PD deficiency

CFTR: cystic fibrosis transmembrane conductance regulator; CYP: cytochrome P450; HLA: human leukocyte antigen; G6PD: glucose-6-phosphate dehydrogenase; HGPRT: hypoxanthine-guanine phosphoribosyltransferase; HIV: human immunodeficiency virus.

**Table 4:** Genes with polymorphisms showing significant association with response to IFN*β* in patients with multiple sclerosis from candidate-gene studies [39].

Gene (polymorphic locus)	Product function (if known)	Number of loci	Nature of response for each polymorphism
*IFNAR1 *(rs1012334; rs55884088) [41, 42]	Type I membrane protein that forms one of the two chains of a receptor for IFN*α* and IFN*β*	2	+, +

*LMP7 *(rs2071543) [42]	Subunit of the immunoproteasome which generates peptides presented on MHC class I molecules to cytotoxic T cells	1	+

*CTSS *(rs1136774) [42]	Cathepsin S, a lysosomal cysteine proteinase that may participate in the degradation of antigenic proteins to peptides for presentation on MHC class II molecules	1	+

*MXA *(rs2071430; rs17000900) [42]	An antiviral protein induced in immune system cells by type I IFNs	2	+, +

*IRF5 *(rs2004640) [43]	IFN regulatory factor 5, transcription factor involved in both type I IFN and Toll-like receptor signaling pathways	1	−

*IRF8 *(rs17445836) [44]	IFN regulatory factor 8, transcription factor of the IFN regulatory factor family	1	−

*USP18 *(rs2542109) [45]	Member of the ubiquitin-specific proteases family of enzymes that cleave ubiquitin from ubiquitinated protein substrates	1	+

*IFNG *(polymorphic microsatellites in the first intron) [46]	IFN*γ*	4	−, −, −, +

*IL10 *(rs1800896; rs1800871; rs1800872) [47]	Interleukin-10	1	+

*CCR5 *(rs333) [48]	Member of the *β* chemokine receptor family, expressed by T cells and macrophages	1	+

*TGFB1 *(rs1800469) [48]	Member of the transforming growth factor *β* family of cytokines	1	+

*TRAILR1* (rs20576) [49]	Tumor necrosis factor-related apoptosis-inducing ligand	1	+

*CD58* (rs12044852) [50]	Ligand of the T lymphocyte CD2 protein and functions in adhesion and activation of T lymphocytes	1	−

*CD46* (rs2724385) [51]	Type I membrane protein that is a regulatory part of the complement system	1	+

*GPC5* (rs10492503; rs1411751) [52]	Cell surface heparan sulfate proteoglycan	2	+, +

+: associated with positive treatment response; −: associated with negative treatment response; CD: cluster of differentiation; IFN: interferon; MHC: major histocompatibility complex.

**Table 5:** Selected genes with polymorphisms showing significant association with response to interferon *β* in patients with MS from genome-wide association studies.

Gene (polymorphic locus)	Product function (if known)
*GPC5* (rs10492503; rs9301789) [54]	Glypicans are implicated in synapse formation and axon regeneration and guidance and are found in dense networks in active MS plaques, where they may be involved in sequestering proinflammatory chemokines

*COL25A1* (rs794143) [54]	Collagen type XXV *α*1, a brain-specific membrane-bound collagen

*HPLN1* (rs4466137) [54]	Hyaluronan proteoglycan link protein 1, an extracellular matrix protein

*CAST* (rs10510779) [54]	Calpastatin, a calpain (calcium-dependent cysteine protease) inhibitor

*NPAS3* (rs4128599) [54]	Neuronal PAS domain protein 3, a member of the basic helix-loop-helix and PAS domain-containing family of transcription factors

*GRIA3* (rs12557782) [56]	*α*-Amino-3-hydroxy-5-methyl-4-isoxazolepropionic acid-type glutamate receptor component

*ADAR* (rs2229857) [56]	Type 1 interferon-related protein, responsible for RNA editing by site-specific deamination of adenosines

*IFNAR2* (rs2248202) [56]	Interferon (*α*, *β*, and *ω*) receptor 2

*CIT* (rs7308076) [56]	Serine/threonine-protein kinase that functions in cell division

*ZFAT* (rs733254) [56]	Zinc-finger protein, binds DNA and functions as a transcriptional regulator involved in apoptosis and cell survival

*ZFHX4* (rs11787532) [56]	Zinc-finger protein, may play a role in neural and muscle differentiation

*STARD13* (rs9527281) [56]	Guanosine triphosphatase-activating protein, may be involved in regulation of cytoskeletal reorganization, cell proliferation, and cell motility

*SLC9A9* (rs9828519) [57]	Solute carrier family 9, subfamily A (NHE9, cation proton antiporter 9), member 9, localized in endosomes and may play an important role in maintaining cation homeostasis

*NINJ2* (rs7298096) [55]	Ninjurin 2, member of the ninjurin (for nerve injury induced) family

*TBXAS1* (rs4726460) [55]	Thromboxane A synthase 1, member of the cytochrome P450 superfamily of enzymes that catalyze many reactions involved in drug metabolism

*GRM3* (rs2237562) [55]	Glutamate receptor 3

*GRIK2* (rs1475919) [55]	Glutamate receptor, ionotropic, kainate 2

*FHIT* (rs760316) [58]	Fragile histidine triad involved in purine metabolism

*GAPVD1* (rs10819043; rs2291858; rs10760397) [58]	Guanosine triphosphatase activating protein and VPS9 domains 1, participates in various processes such as endocytosis, insulin receptor internalization, or LC2A4/GLUT4 trafficking

MS: multiple sclerosis.

**Table 6:** Genes with polymorphisms showing significant association with response to glatiramer acetate in patients with multiple sclerosis [39].

Gene (polymorphic locus)	Product function (if known)	Number of loci	Nature of response for each polymorphism
*HLA-DRB1* (N/A; rs3135388; N/A; N/A) [44, 63–65]	HLA class II MHC antigen, DRB1 *β* chain	4	+, +, +, +
*HLA-DQB1* (N/A) [64]	HLA class II MHC, class II, DQ *β*1	3	+, −, −
*CCR5* (rs333) [65]	C–C chemokine receptor type 5	1	+
*TCRB* (rs71878) [40]	T cell receptor *β* chain	1	+
*IL12RB2* (rs946685) [40]	Interleukin-12 receptor subunit *β*2	1	−
*MBP* (rs470929) [40]	Myelin basic protein	1	+
*IL1R1* (rs956730) [40]	Interleukin-1 receptor type 1	1	+
*CD86* (rs1129055) [40]	CD86 molecule providing costimulatory signals necessary for T cell activation	1	+
*CTSS* (rs2275235; rs1415148) [40]	Cathepsin S	2	+, +
*FAS* (rs982764) [40]	Fas cell-surface death receptor	1	+

+: associated with positive treatment response; –: associated with negative treatment response; CD: cluster of differentiation; MHC: major histocompatibility complex; N/A: not available.
